# A Study on Emotional Intelligence, Breastfeeding Self-Efficacy, and Prenatal Maternal Expectations in Women Attending a Pregnancy School

**DOI:** 10.3390/jintelligence13030035

**Published:** 2025-03-10

**Authors:** Aleyna Bayındır, Hülya Tosun

**Affiliations:** Department of Midwifery, Health Science Faculty, Kütahya Health Science University, Kütahya 43700, Turkey; aleyna.bayindir.ab@gmail.com

**Keywords:** emotional intelligence, antenatal classes, breastfeeding self-efficacy, prenatal maternal expectations, midwifery

## Abstract

This study was conducted to determine the relationship between emotional intelligence (EI), breastfeeding self-efficacy, and maternal expectations of women who did and did not receive education and counseling during pregnancy. An observational cross-sectional study was conducted in a state hospital with 146 pregnant women (intervention group, *n* = 72; control group, *n* = 74). The intervention group had five stages, while the control group received standard pregnancy care. Data is collected by the “Personal Information Form”, “Rotterdam EI Scale”, “Prenatal Breastfeeding Self-Efficacy Scale”, and “Prenatal Maternal Expectations Scale”. When the emotional intelligence scores increased in the intervention group, breastfeeding self-efficacy and antenatal motherhood expectations also increased in the intervention group. In addition, the intervention group’s EI, EI self-evaluation sub-dimension, prenatal motherhood expectations, unrealistic negative motherhood expectations mean, and breastfeeding self-efficacy scale were higher than those of the control group. The regression analysis revealed that the “self-evaluation” sub-dimension of the EI in the intervention group is correlated with regulate others and their own emotions, EI, breastfeeding self-efficacy, and prenatal motherhood expectations. This study shows that pregnant women who attended antenatal classes during the prenatal period had higher EI, breastfeeding self-efficacy, and prenatal maternal expectations than those who were pregnant and did not receive education.

## 1. Introduction

Pregnancy represents an important stage in a woman’s life (Diotaiuti et al. 2022). During this period, women experience a range of physical, hormonal, and psychological changes, which can collectively contribute to feelings of anxiety and stress (Wu et al. 2024). Such anxieties may cause adverse outcomes, including prematurity, low birth weight, and immunosuppression in the fetus during the intrauterine period. These conditions may also refer to an increased risk for the cognitive and social development of the child (Diotaiuti et al. 2022; Hadfield et al. 2022).

Prenatal training has been demonstrated to facilitate the resolution of these issues during the perinatal period, including pregnancy, childbirth, and the postpartum phase. These training programs represent a pivotal component of the World Health Organization’s (WHO) safe motherhood approach (*Güvenli Annelik Katılımcı Kitabı* 2009). Despite the implementation of these training programs, the rates of breastfeeding in the majority of countries remain below the anticipated levels (Kehinde et al. 2023). A report by WHO indicates that the number of exclusively breastfed infants under six months of age worldwide has increased by more than 10% over the past 12 years. This indicates that 48% of infants globally are currently deriving the benefits of this auspicious beginning to their lives (World Health Organization 2024). Factors that prevent the initiation and maintenance of breastfeeding include mastitis, fatigue, the perception of insufficient milk, anxiety about returning to work, low self-efficacy, feelings of insecurity, and psychological problems (Kehinde et al. 2023). It is crucial to provide comprehensive breastfeeding education during the antenatal period, offer tailored support according to the woman’s needs, arrange follow-ups in line with her self-efficacy level in the postpartum period, and give guidance to facilitate the breastfeeding process of mothers in the postpartum period (Durmazoğlu et al. 2024).

The experience of motherhood is associated with several significant challenges for women, including elevated stress levels, fatigue, and an increased workload (Gebhardt et al. 2024). Such responsibilities may cause women to idealize motherhood, distracting them from a positive experience characterized by satisfaction. The image of the “perfect birth” and “perfect mother,” particularly on social media and in popular culture, exerts considerable pressure to conform to this idealized notion. It also leads many women to strive for perfection and to hold unrealistic expectations (Henderson et al. 2016; Tate 2023).

The discrepancy between the reality of motherhood and the ideology that precedes it has been linked to feelings of failure, guilt, and anxiety, which can potentially culminate in depression (Henderson et al. 2016; Rizzo and Watsford 2020; Sonnenburg and Miller 2021). It is, therefore, crucial for women to engage in training programs that provide them with knowledge and skills to navigate the potential challenges they may face during the birth and postpartum period, thereby safeguarding their well-being and that of their infants. Such efforts can enhance maternal perception, ensure preparedness for the birth and postpartum process, and facilitate managing these stages (McCarthy et al. 2021; Almalik and Mosleh 2017; Yuvaci et al. 2021).

The Emotional intelligence (EI) of pregnant women is a significant determinant of their capacity to navigate the antepartum, peripartum, and postpartum periods healthily. It develops as a result of the interaction between emotions and intelligence while it helps individuals to recognize their own and others’ emotions, differentiate between them, and utilize this understanding in practical contexts (Özer and Erkek 2021). EI has a significant effect on maternal behavior (Goleman 1996). Mothers with high EI tend to understand their children’s emotional needs better, and they respond appropriately. Researches show that mothers with high EI levels communicate well and effectively with children and contribute positively to their emotional development (Petrides et al. 2016). It helps parents exhibit emotionally harmonious and appropriate behaviors. In addition, mothers with high EI levels can cope more effectively with stressful situations and approach their children more carefully, caringly, and empathically. In addition, such mothers support their children’s psychological well-being by being more understanding, patient, and fair when disciplining them. They can also remain more emotionally balanced and remain calm in the face of stress (Valadi et al. 2022). 

Daniel Goleman, who introduced the concept of EI, indicates that it comprises the components of self-awareness, self-regulation, social skills, empathy, and motivation (Drigas and Papoutsi 2018).

EI is a multidimensional concept that includes individuals’ abilities to understand, regulate, and use their emotions effectively in social relationships (Salovey and Mayer 1990). In this context, the Trait EI Model developed by Petrides (2001) defines EI by associating it with an individual’s personality traits and differences. This model is built on four basic components of emotion perception, emotion understanding, emotion regulation, and emotion use. The Trait EI model assesses individuals’ perceptions of emotional competence by establishing a strong relationship with the Big Five Model (Petrides et al. 2016). The Rotterdam Emotional Intelligence Scale (REIS), which was developed similarly to the Trait EI model, has a different approach that separately assesses how an individual manages their own and others’ emotions. It is a measurement tool with high psychometric validity and internal consistency that allows for detailed analysis of EI over four basic components (Pekaar et al. 2018). This scale allows for a more comprehensive examination of the individual and social aspects of EI. The main difference between the Trait EI model and REIS stems from the conceptual approaches they address. While the Trait EI model addresses EI as a personality-based structure, REIS offers a more situational and competency-based measurement. It provides the opportunity to analyze both trait-based (trait EI) and ability-based (ability EI) approaches, revealing different aspects of EI. This flexibility makes REIS an applicable measurement tool in various fields such as clinical psychology, organizational psychology, and education.

There is an important relationship between the Pregnancy Education Program and the REIS sub-dimensions used in this study in terms of individual and social learning processes. The Pregnancy Class is a program that provides comprehensive information to expectant mothers about pregnancy, birth, and motherhood through five main education modules. However, these educations are not only based on information transfer but also create an environment that develops EI through individual awareness, social learning, and peer support. In this context, there are important conceptual connections between the four sub-dimensions of the REIS and the contents of pregnancy education.

The impact of EI on the processes of pregnancy, childbirth, and the postpartum period remains an ongoing investigation. EI is a concept that encompasses a range of characteristic features, including empathy, self-efficacy, interpersonal relationships, management skills, and the capacity to cope with problems (Buko and Özkan 2016). It is distinguished from cognitive intelligence (CI) by its focus on emotional and social processes. Consequently, this type of intelligence can be a determining factor in a woman’s capacity to cope with the challenges she may face during pregnancy, childbirth, and the postpartum period (Buko and Özkan 2016). The study of Mammadov and Erenel (2021) noted that mothers with high EI were more successful in fulfilling their maternal roles as they examined the relationship between women’s EI levels and their maternal roles. Furthermore, Özer and Erkek (2021) indicated that a significant correlation existed between women’s EI and their mean on the readiness for childbirth, fear of childbirth, and stress coping scales.

Various studies have shown that there are significant differences in EI between women who attend pregnancy schools and those who do not during the prenatal period. Pregnancy education programs can increase EI levels by offering content that strengthens women’s emotional awareness and coping mechanisms. Previous studies have examined the effects of pregnancy schools and education programs on EI in detail. In a study conducted by González and Fernández (2016), women who attended pregnancy schools and those who did not attend were compared, and significant increases in women who received education were observed. Examining the relationship between EI and birth experience, Kaydırak et al. (2023) stated that the increase in the EI levels of women who received pregnancy education helped them manage the birth process more effectively. Similarly, Özer and Erkek (2021) emphasize that pregnancy schools improve women’s EI levels in the prenatal period and contribute to a more positive birth experience in this process. Women who do not receive pregnancy education generally have more difficulties in emotional regulation and stress management, which may increase their risk of postpartum depression (Mannava et al. 2015). Therefore, prenatal education not only provides physiological preparation but also increases psychological resilience and EI levels.

The objective of this study was to evaluate the relationship between EI, maternal expectations, and breastfeeding self-efficacy among women who attended and did not attend a pregnancy school during the prenatal period. The rationale for focusing on women attending prenatal classes lies in the systematic curriculum that addresses both the physiological and psychological aspects of the pregnancy process, serving as a standardized and structured intervention method for prenatal education. Consequently, such training programs are implemented in a more controlled environment, allowing for a more systematic evaluation of their effects compared to broader parental support programs. Additionally, this study fills a gap in the literature by providing empirical evidence regarding the relationship between EI, breastfeeding self-efficacy, and maternal expectations. Previous research has often examined these variables independently; however, it has not comprehensively assessed the relationship between structured prenatal education and EI.

## 2. Materials and Methods

The study employed a cross-sectional design, comprising an intervention and a control group. It was conducted between 1 December 2022, and 1 April 2023, in the Pregnancy School Unit of a state hospital. The data consisted of pregnant women aged 19–40 years residing in a district in the Marmara region of Turkey and enrolled in the pregnancy school. The required sample size was determined considering the number of pregnant women who attended the pregnancy school every week. The study’s sample size was determined based on an effect size of d = 0.5, with a 95% confidence level (α = 0.05) and a power of 0.90. These parameters were chosen to ensure sufficient statistical power for detecting meaningful differences between the intervention and control groups. Given the study’s observational and cross-sectional nature, a sample size of 60 participants per group was initially planned. However, considering potential attrition and withdrawal, the sample size is expanded to 72 participants in the intervention group and 74 in the control group to maintain the robustness of the findings.

Regarding potential biases, it is acknowledged that self-reported measures, such as the Rotterdam EI Scale, Prenatal Breastfeeding Self-Efficacy Scale, and Prenatal Maternal Expectations Scale, may introduce social desirability bias. However, to mitigate this, participants were informed about the confidentiality of their responses, and standardized scales with established psychometric properties were used. Additionally, as an observational cross-sectional study, the inherent limitation that causality cannot be inferred is recognized, and there may be unmeasured confounders influencing the results.

The study population was selected from pregnant women who were literate, attended pregnancy school, had a single fetus, did not have a high-risk pregnancy, and provided written informed consent. It was approved by the Non-Interventional Clinical Research Ethics Committee of X Health Sciences University on 15 December 2022, with approval number X.

The research was conducted by the ethical standards outlined in the Declaration of Helsinki. Furthermore, the participants were informed that their personal information was protected safely and that they could withdraw from the study at any time they wanted to without providing any explanation.

The following hypotheses were tested in this study:

**H1:**  *There is a difference in EI between women who did and did not attend pregnancy school during the antepartum period*.

**H2:**  *A correlation exists between EI and breastfeeding self-efficacy of women who did and did not attend pregnancy school during the antepartum period*.

**H3:**  *A correlation exists between EI and maternal expectations of women who did and did not attend pregnancy school during the antepartum period*.

**H4:**  *A relationship exists between breastfeeding self-efficacy and maternal expectations of women who did and did not attend pregnancy school during the antepartum period*.

**H5:**  *There is a relationship between EI, breastfeeding self-efficacy, and maternal expectations of women who did and did not attend pregnancy school during the antepartum period and their demographic characteristics*.

### 2.1. Implementation of the Research

A pre-application was conducted with 12 pregnant women who met the inclusion criteria to assess the comprehensibility and applicability of the methodology. The results indicated that it was required to make any change in the content of the forms. The intervention group contained pregnant women who had completed five stages (each stage includes 3 sessions of 40 min) at the pregnancy school. Pregnant women in the intervention group were provided with training based on the Pregnancy Information Classes Training Book presented by the Republic of Turkey Ministry of Health in 2014. The curriculum covers basic topics related to pregnancy and the birth process. These include topics such as reproductive organs and the formation of pregnancy, physiological and psychological changes during pregnancy, nutrition and nutritional support, routine examinations and immunization, common problems and solutions during pregnancy, the birth process, drug-free labor pain management, postpartum care, and newborn care. In addition, postpartum family planning methods are also included in the curriculum (Pregnancy Information Class Education Book 2014). 

The questionnaire forms were administered to both the intervention and control groups at the same gestational age. It took approximately 40 min to fill out the questions in the forms. The control group, who declined to participate in the pregnancy school, received the standard care. Although the study included pregnant women at or after the 16th gestational week, two pregnant women residing in rural areas who intended to spend the winter in the district and wished to attend the pregnancy school were also included even though they were in the seventh week of gestation.

### 2.2. Data Collection Tools

#### 2.2.1. Personal Information Form

The form consists of 10 items to ascertain the socio-demographic and obstetric characteristics of the female participants, as well as their opinions and experiences regarding their involvement in prenatal education.

#### 2.2.2. Prenatal Breastfeeding Self-Efficacy Scale

The scale was developed by Wells et al. (2006) to determine the breastfeeding self-efficacy perceptions of pregnant women in the prenatal period. It comprises a total of 20 items, while its Cronbach’s alpha coefficient was 0.89. The Turkish validity and reliability study of the scale was revised by Aydın and Pasinlioğlu (2018), comprising four sub-dimensions of the skills considering desires for breastfeeding, gathering information to breastfeed, breastfeeding in the presence of other people, feelings during breastfeeding, and social pressure during breastfeeding. It employs a 5-point Likert scale with a minimum value of 20 and a maximum value of 100. The higher amounts indicate an enhanced perception of breastfeeding self-efficacy (Aydın and Pasinlioğlu 2018) with Cronbach’s alpha coefficient of 0.85. In the present study, the Cronbach’s alpha coefficient for the Prenatal Breastfeeding Self-Efficacy Scale (PBSES) was obtained to be 0.893.

#### 2.2.3. Rotterdam Emotional Intelligence Scale

The original version of the Rotterdam Emotional Intelligence Scale (REIS) was developed by Pekaar et al. (2018). Tanrıöğen and Türker (2019) adapted it in Turkish, comprising 28 items and factors of evaluation of one’s own emotions and others’ emotions as well as regulate one’s own emotions and others’ emotions. The lowest value on this five-point Likert-type scale is 28, while the highest one is 140 (Pekaar et al. 2018). The Cronbach Alpha Coefficient of it was 0.94 while it was 0.937 in this study.

#### 2.2.4. Prenatal Maternal Expectations Scale

This scale was developed by Coleman et al. (1999) to assess the mother’s psychological adaptation to the experience of pregnancy and motherhood during the prenatal period.

The Turkish version of the scale was adapted by Şendil et al. (2016), comprising two sub-dimensions and 34 items. A five-point Likert-type scale is employed with a minimum value of 34 and a maximum of 170. High values indicate the presence of unrealistic positive expectations, whereas low values point to the presence of unrealistic negative expectations (Şendil et al. 2016).

In the reliability study of the Prenatal Maternal Expectations Scale (PMES), the Cronbach’s alpha coefficient for the unrealistic positive expectations sub-dimension was determined to be 0.95, while it was 0.87 for the unrealistic negative expectations sub-dimension (Şendil et al. 2016). In this study, Cronbach’s alpha coefficient was determined to be 0.837 for the unrealistic negative expectations subscale and 0.892 for the unrealistic positive expectations subscale.

### 2.3. Statistical Analysis

The IBM SPSS Statistics 22 program (SPSS Inc., Chicago, IL, USA) was employed for the analysis of the data obtained from the 146 participants. The scale scores of a participant were excluded from the study due to the presence of extreme values. The results were evaluated by considering 72 participants in the intervention and 74 participants in the control group. The skewness and kurtosis values of the scale were examined while parametric methods were employed in comparisons related to scale scores since the normality assumption was met. In the analysis of the data, descriptive categorical data were presented as number (*n*) and percentage (%), while quantitative data were presented as mean and standard deviation values as well as skewness, kurtosis, minimum, and maximum values. The independent-sample *t*-test was employed to ascertain whether there were any significant differences in the means of the two independent groups. Pearson correlation analysis was employed to investigate the relationship between scale scores and quantitative demographic variables. A *p*-value of less than 0.05 was considered statistically significant. Regression analysis was also used to investigate the influence of a single independent variable on multiple dependent variables.

## 3. Results

The demographic information about the participants is provided in Table 1. Most of the participants were university graduates, with income equal to expenditure, and primiparous pregnant women.

The statistics in Table 1 represent the demographic information of women who attended and did not attend pregnancy school. It represents results whether there is a significant difference between the demographic characteristics or they are independent. A significant relationship was determined between the educational backgrounds of women who attended and did not attend pregnancy school. Accordingly, it was observed that 65.3% of those with university and higher education attended pregnancy school, while 44.6% did not attend pregnancy school. No significant difference was obtained between the comparisons of other parameters (*p* > 0.05). Accordingly, the results show that the groups are independent, and there is no difference between them in terms of demographic variables.

The statistics in Table 2 represent the mean REIS total of the women who attended the pregnancy school was 103.76, while it was 98.97 for those who did not. The mean of the breastfeeding self-efficacy scale of the women was 88.23 and 81.72 for the mentioned groups. The mean of the prenatal maternity expectations scale for women who did and did not attend the pregnancy school were 141.94 and 137.94, respectively.

Table 3 represents the statistical results of the comparison of scales of women who did and did not receive prenatal education during pregnancy. A notable discrepancy was observed between the PBSES totals of the two groups (*p* < 0.05). The results showed that the breastfeeding self-efficacy of women who had attended the pregnancy school was higher than those who did not.

Also, a significant difference was observed between PMES and the unrealistic negative expectations of those who did and did not attend pregnancy school (*p* = 0.047 < 0.05). Consequently, the unrealistic negative expectations mean was higher for those who attended the school.

Table 4 represents the statistical results of the participants’ education. A significant difference was observed between the aggregate of the intervention and control groups with secondary education and below regarding REIS, other focused emotion-appraisals (OFEA), self-focused emotion- regulation (SFER), and PBSES (*p* < 0.05). Among women with secondary education or less, those who attended pregnancy school demonstrated higher values in the evaluation of others’ emotions, emotional control, EI, and breastfeeding self-efficacy than those who did not attend the school.

There was no significant difference in REIS SFEA scores between women with secondary education or below who went to pregnancy school and those who did not go to pregnancy school (*p* > 0.05).

A significant difference was determined in REIS OFEA scores between women with secondary education or below who attended pregnancy school and those who did not attend pregnancy school (*p* < 0.05). It was obtained that women with secondary education or below who went to pregnancy school were more able to evaluate the feelings of others and control their feelings than women who did not go to pregnancy school.

A significant difference was obtained in total PBSES scores between women with secondary education or below who went to pregnancy school and those who did not go to pregnancy school (*p* < 0.05). Accordingly, among women with secondary education or below who went to pregnancy school, breastfeeding self-efficacy scores of those who went to pregnancy school were higher than those of women who did not go to pregnancy school.

Correlation and *p*-values between scales and some demographic characteristics of women who received prenatal education are provided in Table 5. A significant and positive correlation was determined between the Rotterdam Self-Focused Emotion-Appraisal (RSFEA) and other scales, including Rotterdam Other Focused Emotion-Appraisal (ROFEA), SFER, Other Focused Emotion-Regulation (OFER), and totals when the women attended the pregnancy school were evaluated (*p* < 0.05). This finding indicates the women’s capacity to assess their own emotions to improve, including their comprehension of others’ emotions, EI, confidence in their ability to breastfeed, and their expectations regarding the prenatal period. A similar correlation was found between the RSFEA and OFER as well as EI. In addition, a positive and significant correlation was determined between the Rotterdam OFER and EI, PBSES, and PMES (*p* < 0.05). The results showed women’s ability to control others’ emotions and EI with the increasing ability to control their own emotions.

For the women who attended the pregnancy school, a moderate positive correlation (*p* < 0.05) was determined between the scales of PBSES and PMES, which indicates that an increase in breastfeeding self-efficacy would also increase prenatal expectations.

On the other hand, a weak positive correlation was determined between the PMES unrealistic positive expectancy and the RSFEA, OFEA, OFER, and EI. Conversely, positive correlations were observed between unrealistic negative expectancies and the aforementioned variables. These findings indicate that emotional-appraisal and expectations during pregnancy are interrelated.

Pearson correlation analysis was performed to examine the relationship between some demographic characteristics of women who received prenatal education and counseling and scale scores. A positive, weak relationship was found between the number of pregnancies and live births and regulate one’s own emotions (*p* < 0.05). Accordingly, it was determined that as the number of pregnancies and live births increased in women who attended pregnancy school, regulate one’s own emotions also increased. No significant relationship was found between the REIS SFER score of women who attended pregnancy school and their age and how many weeks pregnant they were (*p* > 0.05).

Table 6 represents the correlation and *p*-values between scales and some demographic characteristics of women who did not receive prenatal education. Among women who had not attended pregnancy school, no statistically significant relationship was identified between Rotterdam OFEA and age, number of pregnancies, and number of live births (*p* > 0.05). Nevertheless, a significant and positive correlation was observed between the Rotterdam OFEA and gestational week (*p* < 0.001), indicating the capacity to regulate the emotions as gestational age increases.

No significant correlation was determined between the PMES and either women’s age or gestational week (*p* > 0.05). However, a significant negative correlation was observed between the number of pregnancies and live births and the PMES (*p* < 0.05, *p* < 0.001). Consequently, the increase in the number of live births and pregnancies among women who did not attend pregnancy school is linked to a decline in prenatal maternal expectations.

Table 7 represents the correlation and *p*-value statistics of women who received prenatal education. Among the women who participated in the pregnancy school, statistically significant positive correlations were observed between the RSFEA and the others (ROFEA, SFER, OFER, totals, PBSES, PMES) at the 0.05 level. Such a result suggests that individuals’ EI, confidence in their ability to breastfeed, and expectations regarding their prenatal experience as a mother increase with their capacity to assess emotions effectively. Furthermore, a positive and significant correlation was observed between the Rotterdam SFER and the OFER, as well as EI (*p* < 0.05). However, no significant correlation was found between the PBSES and PMES (*p* > 0.05).

The Rotterdam OFER was determined to exhibit a positive and significant correlation with EI, PBSES, and PMES (*p* < 0.05). In addition, the positive and significant correlations between them indicate that prenatal motherhood expectations increase with breastfeeding self-efficacy.

A weak positive correlation was identified between the PMES unrealistic positive expectancy and RSFEA, OFEA, OFER, and EI (*p* < 0.05). It was determined that the level of breastfeeding self-efficacy increases with unrealistic positive expectations. Furthermore, weak positive correlations were observed between the unrealistic negative expectancy of PMES and RSFEA as well as EI (*p* < 0.05).

The correlation and *p*-values of the women who did not receive prenatal education are presented in Table 8. For the women who had not attended a pregnancy school, a positive and significant relationship was observed between the RSFEA and others (OFEA, SFER, OFER, totals, and PBSES) (*p* < 0.05). However, no significant relationship was obtained between the RSFEA and the PMES (*p* > 0.05). This indicates that the assessment of others’ emotions, emotional control, and breastfeeding self-efficacy increases with the assessment of one’s own emotions. Additionally, positive and significant relationships were identified between the ROFEA and the SFER, OFER, and EI (*p* < 0.05).

No correlation between PMES–maternal age and PMES–gestational duration was identified, but a negative correlation was observed between the number of pregnancies and the number of live births (*p* < 0.05, *p* < 0.001). On the other hand, no significant correlation was determined between PMES unrealistic positive expectancy and others, while positive correlations were observed between the unrealistic negative expectancy and others.

According to the regression analysis, it was observed that regulate others’ and one’s own emotions, EI, breastfeeding self-efficacy, and prenatal motherhood expectations are parallel with “evaluating own emotions” in the intervention group (*p* < 0.05).

## 4. Discussion

Holistic training given in childbirth preparation classes is important for the protection and development of maternal and infant health. In this section, the discussion of the study is provided under three headings to ensure the integrity of the subject.

Comparison of Emotional Intelligence of Women with and without Prenatal Education and Counseling:

The complex maternal role presents a significant physical, mental, and emotional challenge for women. To achieve success in these roles, women require the assistance of support systems. EI, which is an inherent capacity along with CI and can be cultivated over time, is one of the support systems that enables women to cope with difficulties and identify rational solutions. Women with a high level of EI are better able to assess their health status, solve problems more effectively, increase their self-motivation, and focus their attention on emotion (Kaydırak et al. 2023). The most significant factor in the advancement of EI is education (Gilar-Corbi et al. 2019; Mattingly and Kraiger 2019; Opatha and Takahashi 2024).

The results of this study indicate that women who attended the pregnancy school exhibited higher EI than those who did not. This finding supports the H1 hypothesis of the study. It was revealed that woman who attended the pregnancy school exhibited superior self-assessment of their emotional states. Buko and Özkan (2016) examined the EI and prenatal attachment status of pregnant women and observed that the EI of pregnant women was at average levels. Additionally, the sub-dimension of expressing their own emotions had a higher score than the others, as evidenced in this study. Ebrahimi et al. (2014) evaluated the EI and postpartum depression levels of women who had undergone either a normal delivery or a cesarean section. They found that there was no significant difference between the EI of the two groups of women. However, they reported that “self-awareness” was the sub-dimension with the highest score among the EI sub-dimensions. The studies showed that emotional awareness is associated with several beneficial outcomes, including enhanced emotional management, the capacity to respond effectively in complex social situations, satisfaction from relationships, and improved physical and mental health (Lane and Smith 2021). In this context, it is of the utmost importance for pregnant women to possess high levels of emotional awareness, cultivate positive emotions during pregnancy, adapt to the new family structure, meet the needs of their infant effectively, and fulfill their roles while maintaining their freedom.

This study’s results showed that the women’s ability to control their own emotions, evaluate and control others’ emotions, EI, breastfeeding self-efficacy, prenatal motherhood expectations, and the evaluation of their own emotions increased if they attended pregnancy school. It shows that it supports hypotheses H1, H2, and H3. The ability to understand one’s own emotions and intentions is a fundamental prerequisite for accurately perceiving the emotions of others. Approaches such as the “Theory of Mind” emphasize that this awareness fosters empathy and contributes to the formation of stronger interpersonal relationships (Gallagher and Frith 2003; Morin 2011; Silvestri et al. 2024). Moreover, self-awareness is associated with EI, which in turn increases an individual’s ability to understand others’ emotions and respond appropriately (Goleman 2001; Ashkanasy and Dasborough 2003; Côté 2017). This indicates that individuals who possess self-awareness tend to enhance their social competencies.

The findings in this study support that, indicating that the observed increase in EI and self-efficacy levels among women attending pregnancy school also affects their general self-control abilities. The capacity to assess one’s own emotions, discern one’s desires from one’s aversions, and exercise self-control is a personal attribute that can enhance one’s capacity to regulate the emotions of others (Carpinelli and Savarese 2022). The results indicate that pregnant women develop EI and self-efficacy components to navigate the prenatal and postnatal periods with optimal efficacy.

Prenatal maternal expectations may influence EI and breastfeeding self-efficacy. The mother’s expectations may exert a positive or negative influence on the breastfeeding process and the adoption of the parenting role. A comprehension of the interrelationship between these variables may assist women to better prepare for childbirth and motherhood to facilitate more favorable parenting experiences.

The results of this study indicate that there was an increase in both the breastfeeding self-efficacy scale and the PMES as the REIS increased among women who attended the pregnancy school. It supports the H1 hypothesis of the study. The study conducted by Mammadov and Erenel (2021) concluded that mothers with high EI fulfilled their maternal roles more successfully. The findings in this study are parallel to this study. Özer and Erkek (2021) researched the EI of pregnant women and their preparedness for childbirth, stress, and fear of childbirth. They indicated that when pregnant women exhibited low EI, they demonstrated lower levels of childbirth preparedness and higher levels of fear. The results of this study and the literature suggest that as women’s EI varies, their breastfeeding self-efficacy, maternal roles, and expectations of the birth process may vary in the same direction.

In this case, training programs that address stress management, role transition, communication, and emotional awareness during pregnancy, childbirth, and the postpartum period, as offered in pregnancy schools, have the potential to positively influence women’s EI. It is known that EI is amenable to enhancement through educational interventions (Kotsou et al. 2019). Nevertheless, it is essential to take individual differences into account when generalizing these results.

The result of this study indicates a positive and statistically significant correlation (*p* < 0.05) between the REIS, PBSES (r:0.475), and the expectations of motherhood before birth (r:0.285) for the women who did not attend the pregnancy school. These results support the hypotheses of H1 and H3. Accordingly, it is postulated that family interactions, social and cultural structure (Crowne 2009), life experiences, and social support systems may positively influence EI and maternal expectations (Tuxunjiang et al. 2023).

Comparison of Prenatal Breastfeeding Self-Efficacy of Women with and without Prenatal Education and Counseling:

The results of this study indicate that women who attended a pregnancy school exhibited higher breastfeeding self-efficacy scores than those who did not attend the program. This finding lends support to the H1 hypothesis proposed in this study. Corby et al. (2021) identified the predictors of prenatal breastfeeding self-efficacy. Their findings indicated that prenatal education had a significant impact on breastfeeding self-efficacy. Additionally, Özdemir et al. (2022) investigated breastfeeding self-efficacy in adolescent pregnant women and found that the same result was evident in adolescents who received education, while Öztürk et al. (2022) observed that women who received training exhibited enhanced perceptions of breastfeeding self-efficacy and demonstrated greater success in breastfeeding in the initial postpartum week compared to those who received standard care.

Breastfeeding self-efficacy is influenced by many factors, such as the mother’s level of knowledge, past experiences, and the physical and emotional support she receives (Piro and Ahmed 2020; Shafaei et al. 2020; Bülbül and Menekşe 2024). However, EI is also identified as a significant predictor of the mother’s attitudes and behaviors during breastfeeding (Haghighi and Abbasi 2015). Mothers with high EI can be more resilient in coping with challenging situations, can understand their infants’ signals better and respond appropriately, and can increase their self-confidence. Therefore, EI can be considered as an element of support that positively affects both the physical and psychological aspects of the breastfeeding process.

The results of this study indicate that the breastfeeding self-efficacy of the women increased as their EI increased (*p* < 0.05). This finding lends support to the H1 hypothesis proposed in the study. Arshadi Bostanabad et al. (2024) discovered a statistically significant positive correlation between EI and breastfeeding self-efficacy among women with preterm infants. On the other hand, Karakoç et al. (2020) investigated the relationship between EI and breastfeeding self-efficacy in the early postpartum period and did not identify a correlation between these variables. The discrepancies between the findings of these two studies may be attributed to the dissimilar characteristics of the examined samples. Conversely, it is difficult to assert that an increase in breastfeeding self-efficacy is a direct consequence of an increase in EI. However, the findings in this study indicate that EI is one of the factors affecting the mother’s breastfeeding experience.

The other findings of this study indicated that prenatal motherhood expectations were increased with breastfeeding self-efficacy increase among women who attended the pregnancy school (*p* < 0.05). The involvement of expectant mothers in pregnancy schools is significant in terms of acquiring knowledge about childbirth and breastfeeding, developing practical abilities, exchanging experiences, and obtaining emotional assistance (Mueller et al. 2020). As a result of this participation, women may feel more informed, prepared, and secure. Such experiences can facilitate the processes of childbirth and parenting. Pregnant women believe that breastfeeding will be easier and more satisfying after the birth since they will be better equipped to meet the needs of the infant (Öztürk et al. 2022; Kakaşçı et al. 2023).

McGovern et al. (2023) reported that prenatal breastfeeding self-efficacy is shaped by a woman’s expectations during the prenatal period and is affected by her self-confidence and confidence in her perceived ability to breastfeed. In Hankel et al. (2019), the maternal self-efficacy, breastfeeding self-efficacy, and breastfeeding experiences of a group of women at 32 weeks of pregnancy and 3 months after birth were examined. It was found that women who demonstrated high levels of breastfeeding self-efficacy during pregnancy exhibited enhanced self-efficacy in the postpartum period. This result indicates that a focus on enhancing prenatal self-efficacy may lead to enhanced breastfeeding outcomes. However, the mothers may experience stress and anxiety disorders if they develop excessive or unrealistic expectations when it is crucial for them to be adequately prepared for childbirth and motherhood (Adams et al. 2021). Therefore, having realistic expectations can increase expectant mothers’ readiness for the challenges of the birth event and motherhood.

This study revealed that pregnant women with secondary education levels and below had higher breastfeeding self-efficacy compared to those who did not attend a pregnancy school (*p* < 0.05). This finding supports the H5 hypothesis proposed in the study. In the meta-analysis conducted by Brandão et al. (2018), women with higher education levels exhibited lower breastfeeding self-efficacy. This result demonstrated that a positive correlation between maternal education level and breastfeeding self-efficacy is not consistently evident, as observed in this study. There are several potential explanations for this phenomenon. For example, the assumption that educated mothers possess sufficient knowledge about infant care may lead to difficulties in the breastfeeding process and prevent them from seeking support. Furthermore, it is established that highly educated women are more likely to engage in business activities, which may result in challenges during the breastfeeding process (Lechosa-Muñiz et al. 2021). Furthermore, the tendency towards perfectionism, which is prevalent among highly educated women, can precipitate stress and anxiety during the postpartum period as it negatively impacts breastfeeding self-efficacy (Elder et al. 2021) and may result in a cessation of breastfeeding (Segura-Pérez et al. 2022). Therefore, higher education levels can be considered a complex combination of factors affecting breastfeeding self-efficacy. In this context, it is recommended to develop more inviting and supportive educational approaches to support mothers’ breastfeeding experiences.

Tokat et al. (2010) demonstrated that breastfeeding self-efficacy exhibited an increase with education level, but it is inconsistent with the findings of this study. As indicated by data from the Turkish Statistical Institute, the proportion of women in the labor force increased from 27.6% to 35.8% between 2010 and 2023. For this reason, breastfeeding self-efficacy levels may decrease with an increasing labor force participation rate (Febriansyah et al. 2023). Furthermore, research has demonstrated that an individual’s occupational status and the policies governing maternity leave also influence their breastfeeding strategies (de Lauzon-Guillain et al. 2019; Steurer 2017). Breastfeeding is a practice closely linked to cultural values. Research reveals the way breastfeeding practices are shaped by cultural values and how they vary in different societies (Victora et al. 2016; Aygör and Düdükcü 2025; Runjić Babić 2020).

These findings underscore the necessity of fostering a supportive breastfeeding culture and enhancing awareness of maternity rights in Turkey.

Comparison of Maternal Expectations of Women Who Received and Did Not Receive Prenatal Education and Counseling:

In this study, the maternal expectations of women who attended the pregnancy school were determined to be higher than those of women who did not attend the school. In Fasanghari and Keramat (2023), educational interventions were examined regarding maternal competence, and the results indicated that training during and after pregnancy led to a 3.51-unit increase in self-efficacy among primiparous mothers in comparison to the control group. In addition, Talebi et al. (2023) reported that the education received by primiparous women in parenthood preparation classes was about to increase maternal role competence by 3.31 units in comparison to the group receiving routine care. The results of these studies demonstrate that prenatal education programs are an effective means of enhancing maternal self-efficacy.

The result of this study indicates that women attending antenatal classes have more unrealistic positive expectations than those who did not attend such a program. An analysis of the underlying causes suggests that women who attend pregnancy schools tend to gain comprehensive knowledge about childbirth, breastfeeding, infant care, and maternal roles through structured educational programs. It may contribute to a sense of heightened preparedness and confidence in their abilities. Conversely, social media and the reflection of societal norms about the idealized role of motherhood have also been identified as contributing factors to the emergence of these expectations (Mihelic et al. 2016). It is important to note that unrealistic positive expectations may have unintended consequences. The gap between expectations and reality can lead to disappointment and increased stress, especially when plans do not unfold as expected (Kahalona et al. 2022). Therefore, women should be supported to have realistic expectations in prenatal education.

In this study, the women who did not attend a pregnancy school also exhibited more negative and unrealistic expectations before birth. The study by Gress-Smith et al. (2013) on Mexican-American women evaluated prenatal expectations according to cultural characteristics. The results indicated that women who had lower expectations about the fulfillment of the maternal role initiated prenatal care later in their pregnancies. While this result does not entirely align with the findings of this study, it does indicate a comparable general trend. It is hypothesized that women with negative expectations refrain from seeking prenatal care or education about motherhood, as it is attributed to a lack of knowledge, social environment, cultural beliefs, and individual experiences. Individuals tend to fear unknown situations (such as childbirth, breastfeeding, and infant care), and this apprehension is often rooted in a lack of sufficient information and knowledge about these topics. In the context of the role of motherhood, women may develop unrealistic negative expectations towards this unknown role (Mazidi et al. 2024). In addition, the pressure created by the idealized mother model and motherhood norms idealized by society and the media, and previous stressful or challenging experiences, may also negatively affect these expectations.

A closer examination of the reasons for women’s low demand for prenatal education or postponement of these demands reveals the underlying factors contributing to negative maternal expectations. It, therefore, indicates the importance that women benefit more from prenatal education and support services as well as develop more realistic expectations to reduce negative expectations.

The present study revealed a significant correlation between the number of pregnancies (r = −0.347) and the number of live births (r = −0.256) of women who did not attend a pregnancy school and their expectations of motherhood before birth. It can thus be concluded that the expectations of pregnant women decrease as the number of live births and the number of pregnancies increase in women who have not attended a pregnancy school. This finding supports the H5 hypothesis proposed in the study. The study of Gress-Smith et al. (2013) evaluated prenatal expectations from a cultural perspective and concluded that women with high prenatal motherhood expectations tend to have fewer children. The findings in this study are parallel with the results of the present study. The relationship between the number of live births and the number of pregnancies and prenatal maternal expectations can be interpreted from a variety of perspectives. For instance, negative experiences with one’s parents (George and Solomon 2008); traumatic events in the previous pregnancy, birth, or postpartum period; stress, fatigue, and increased responsibility experienced by mothers who have more than one child can lead to the development of negative maternal expectations in women (Zaki et al. 2020). Conversely, an increase in the number of live births is associated with the development of more realistic and balanced expectations in women. The role of motherhood, in which women idealize or have excessive expectations for their first child, becomes increasingly realistic and feasible as they gain experience. Accordingly, to gain a deeper comprehension of the expectations and experiences of mothers about the maternal role, it is feasible to offer suitable assistance through a comprehensive assessment tailored to the individual.

## 5. Conclusions

This study has several limitations. First of all, the data is limited to pregnant women admitted to a single public hospital, which complicates the generalizability of the findings to other groups. To enhance external validity, it is recommended that a larger and more diverse demographic sample be employed. The inclusion of women attending and not attending prenatal classes may present a limitation in terms of sample loss and time management constraints. Furthermore, the assessment of various components, such as breastfeeding self-efficacy, maternal expectations, prenatal education, and EI, necessitates complex interactions, suggesting that a multidimensional approach to interpretation may be required.

This study aimed to determine the relationship between EI, breastfeeding self-efficacy, and maternal expectations among women receiving education and counseling during pregnancy. The findings underscore the importance of prenatal education classes and evidence-based pregnancy school training in enhancing pregnant women’s EI, breastfeeding self-efficacy, and positive maternal expectations. The research indicates that the group receiving education scored higher in terms of EI, the self-assessment sub-dimension of EI, prenatal maternal expectations, unrealistic negative maternal expectations, and breastfeeding self-efficacy compared to the control group. However, the lack of sufficient research on EI during pregnancy has hindered adequate comparisons in the discussion section.

It is crucial for future studies to comprehensively address these issues to strengthen the findings. In particular, further research on the sub-dimensions of prenatal maternal expectations, specifically unrealistic positive and negative expectations, will enhance the generalizability of the results. Incorporating EI-focused content into parenting education programs may serve as an effective strategy for improving maternal health. Systematic instruction of EI will support both maternal health and the healthy development of children.

Future research should investigate the long-term effects of prenatal programs that include EI training on maternal mental health, child development, and parenting styles. Comparative studies considering socioeconomic and cultural differences will help us understand how the effects of prenatal education vary across different contexts. Additionally, to better comprehend the biological underpinnings of this integrated training, it is recommended that studies utilizing biological markers, such as cortisol, be prioritized.

Finally, the integration of EI training into standard prenatal care guidelines can support mothers in coping with emotional challenges, thereby improving overall health and well-being. Providing EI education through digital tools for women unable to physically attend pregnancy schools may offer an effective solution for reaching a broader audience. The incorporation of EI training into health policies will enhance the positive impacts on maternal and child health, contributing to public health.

## Figures and Tables

**Table 1:** Demographic characteristics of participants.

Variables	Categories	Attending the Pregnancy School		Not Attending the Pregnancy School	
		*n*	%	*n*	%
Education Status	Literate	-	-	1	1.4
	Primary school	1	1.4	3	4.1
	Secondary school	6	8.3	13	17.6
	High school	18	25	24	32.4
	Undergraduate or higher	47	65.3	33	44.6
Fisher Exact Test = 7.392, *p* = 0.026					
Spent most of his time	Village	8	11.1	3	4.1
	District	40	55.6	48	64.9
	City	24	33.3	23	31.1
Fisher Exact Test = 2.915, *p* = 0.225					
Income Status	Income less than expenditure	12	16.7	13	17.6
	Income equal to expenditure	48	66.7	45	60.8
	Income higher than expenditure	12	16.7	16	21.6
Fisher Exact Test = 0.700, *p* = 0.701					
Is pregnancy desired or planned	Yes	59	81.9	60	81.1
	No	13	18.1	14	18.9
Pearson Ki Kare test = 0.018, *p* = 0.893					
Having regular pregnancy follow-ups (at least 4 follow-ups) during pregnancy	Yes	70	97.2	71	95.9
	No	2	2.8	3	4.1
Fisher Exact Test = 0.180, *p* = 1.000					
Having any health problems during pregnancy	None	36	50	39	52.70
	Vaginal infection	2	2.78	8	10.81
	Vaginal thrush	3	4.17	1	1.35
	Urinary tract infection	14	19.44	14	18.92
	Hypertension	1	1.39	1	1.35
	Diabetes in pregnancy	5	6.94	4	5.41
	Other	11	15.28	7	9.46
Fisher Exact Test = 9.448, *p* = 0.173					
Age	Mean ± SD	28.29 ± 4.44		28.33 ± 4.33	
	Minimum	19		20	
	Maximum	40		41	
	t = 0.984 *p* = 0.327				
Current number of weeks in pregnancy	Mean ± SD	26.43 ± 10.18		24.61 ± 9.03	
	Minimum	7		8	
	Maximum	40		40	
	t = 1.145, *p* = 0.254				
Number of Pregnancy	Mean ± SD	1.69±1.02		1.77±1.03	
	Minimum	1		1	
	Maximum	5		5	
	t = −0.448, *p* = 0.655				
Number of Live Births	Mean ± SD	0.51± 0.80		0.60±0.83	
	Minimum	.0		.0	
	Maximum	3.0		4.0	
	t = −0.598, *p* = 0.551				

**Table 2:** Descriptive statistics for scale scores.

Scales and Subscales	Attending the Pregnancy School					Not Attending the Pregnancy School				
	Mean ± SS	Min	Max	Skewness	Kurtosis	Mean ± SS	Min	Max	Skewness	Kurtosis
REIS SFEA	29.26 ± 4.75	14	35	−0.582	0.015	28.17±4.57	14	35	−0.448	0.206
REIS BDD	26.16 ± 5.13	15	35	0.036	−0.618	24.91 ± 5.27	13	35	0.177	−0.278
REIS OFEA	24.44 ± 5.60	13	35	0.318	−0.534	22.82 ± 5.95	7	35	0.001	−0.031
REIS BDK	23.88 ± 5.44	13	35	0.193	−0.376	23.05 ± 5.61	12	35	0.468	−0.048
REIS Total	103.76 ± 16.09	76	140	0.172	−0.656	98.97 ± 16.90	63	140	0.456	0.027
PBSES Total Score	88.23 ± 10.24	52	100	−1.224	1.903	81.72 ± 12.37	50	100	−0.529	−0.065
PMES Total Score	141.94 ± 14.10	102	168	−0.381	0.036	137.94 ± 16.62	79	169	−0.998	1.791
PMES unrealistic positive expectations	90.95 ± 9.22	61	105	−0.737	0.524	89.60 ± 12.15	39	105	−1.597	0.552
PMES unrealistic nagative expectations	50.98 ± 7.51	36	65	−0.182	−0.688	48.33 ± 8.42	28	65	−0.475	−0.092

Independent sample *t* test.

**Table 3:** Comparison of categories of women who received prenatal education during pregnancy and those who did not.

Scale and Subscales	Group	*n*	Mean ± SD	*t*	*p*
REIS SFEA	Attending the pregnancy school	72	29.26 ± 4.75	1.408	0.161
	Not attending the pregnancy school	74	28.17 ± 4.57		
REIS OFEA	Attending the pregnancy school	72	26.16 ± 5.13	1.448	0.150
	Not attending the pregnancy school	74	24.91 ± 5.27		
REIS SFER	Attending the pregnancy school	72	24.44 ± 5.60	1.691	0.093
	Not attending the pregnancy school	74	22.82 ± 5.95		
REIS OFER	Attending the pregnancy school	72	23.88 ± 5.44	0.912	0.363
	Not attending the pregnancy school	74	23.05 ± 5.61		
REIS Total	Attending the pregnancy school	72	103.76 ± 16.09	1.753	0.082
	Not attending the pregnancy school	74	98.97 ± 16.90		
PBSES Total	Attending the pregnancy school	72	88.23 ± 10.24	3.455	0.001
	Not attending the pregnancy school	74	81.72 ± 12.37		
PMES Total	Attending the pregnancy school	72	141.94 ± 14.10	1.565	0.120
	Not attending the pregnancy school	74	137.94 ± 16.62		
PMES Unrealistic Positive Expectations	Attending the pregnancy school	72	90.95 ± 9.22	0.755	0.452
	Not attending the pregnancy school	74	89.60 ± 12.15		
PMES Unrealistic Negative Expectations	Attending the pregnancy school	72	50.98 ± 7.51	2.003	0.047
	Not attending the pregnancy school	74	48.33 ± 8.42		

Independent sample *t*-test.

**Table 4:** Comparison of the means of the categories for secondary and lower education (supply doc.).

Scales	Group	*n*	Mean Order Number	U	*p*
REIS SFEA	Attending the pregnancy school	7	14.43	46.00	0.418
	Not attending the pregnancy school	17	11.71		
REIS OFEA	Attending the pregnancy school	7	17.07	27.5	0.040
	Not attending the pregnancy school	17	10.62		
REIS SFER	Attending the pregnancy school	7	18.21	19.5	0.009
	Not attending the pregnancy school	17	10.15		
REIS OFER	Attending the pregnancy school	7	14.64	44.5	0.335
	Not attending the pregnancy school	17	11.62		
REIS total score	Attending the pregnancy school	7	15.93	35.5	0.127
	Not attending the pregnancy school	17	11.09		
PBSES total score	Attending the pregnancy school	7	18	21.00	0.014
	Not attending the pregnancy school	17	10.24		
PMES Total Score	Attending the pregnancy school	7	14.43	46.00	0.391
	Not attending the pregnancy school	17	11.71		
PMES Unrealistic Positive Expectations	Attending the pregnancy school	7	14.57	45.00	0.357
	Not attending the pregnancy school	17	11.65		
PMES Unrealistic Negative Expectations	Attending the pregnancy school	7	12.64	58.5	0.949
	Not attending the pregnancy school	17	12.44		

Independent sample *t*-test.

**Table 5:** Correlation (r) and *p*-values between categories and some demographic characteristics of women who received prenatal education.

Age			Number of Pregnancies	Number of Live Births	Gestational Age
Scales and Subscales					
Rotterdam SFEA	r	−0.073	0.090	0.130	0.061
	*p*	0.541	0.453	0.278	0.612
Rotterdam OFEA	r	−0.091	−0.025	0.037	0.085
	*p*	0.448	0.834	0.758	0.479
Rotterdam SFER	r	−0.118	0.326	0.370	0.215
	*p*	0.323	0.005	0.001	0.070
Rotterdam OFER	r	−0.156	−0.039	0.023	0.085
	*p*	0.191	0.743	0.849	0.478
Rotterdam Total	r	−0.145	0.119	0.187	0.148
	*p*	0.226	0.321	0.116	0.213
PBSES Total	r	0.044	0.007	0.009	0.015
	*p*	0.712	0.953	0.940	0.901
PMES Total	r	−0.086	0.004	−0.053	0.084
	*p*	0.472	0.975	0.657	0.482
PMES Unrealistic Positive Expectations	r	−0.004	0.044	−0.005	0.084
	*p*	0.970	0.715	0.969	0.484
PMES Unrealistic Negative Expectations	r	−0.156	−0.047	−0.094	0.055
	*p*	0.190	0.697	0.431	0.645

Pearson correlation analysis, *p* < 0.05, *p* < 0.001.

**Table 6:** Correlation (r) and *p*-values between categories and some demographic characteristics of women who did not receive prenatal education.

Age			Number of Pregnancy	Number of Live Births	Weeks/Months Number in Pregnancy
Scales and Subscales					
Rotterdam SFEA	r	0.139	−0.023	0.110	−0.045
	*p*	0.237	0.844	0.352	0.701
Rotterdam OFEA	r	0.116	−0.059	0.005	0.306
	*p*	0.324	0.617	0.967	0.008
Rotterdam SFER	r	0.070	−0.040	0.172	0.081
	*p*	0.555	0.734	0.143	0.493
Rotterdam OFER	r	0.033	0.047	0.087	0.072
	*p*	0.780	0.689	0.459	0.542
Rotterdam Total	r	0.110	−0.023	0.121	0.136
	*p*	0.353	0.844	0.305	0.249
PBSES Total	r	0.050	−0.079	−0.109	−0.026
	*p*	0.674	0.502	0.357	0.829
PMES Total	r	−0.184	−0.347	−0.256	−0.098
	*p*	0.116	0.002	0.028	0.408
PMES Unrealistic Positive Expectations	r	−0.234	−0.317	−0.264	−0.063
	*p*	0.045	0.006	0.023	0.592
PMES Unrealistic Negative Expectations	r	−0.026	−0.228	−0.124	−0.101
	*p*	0.823	0.050	0.294	0.392

Pearson correlation analysis, *p* < 0.05, *p* < 0.001.

**Table 7:** The correlation (r) and *p*-values of women who received prenatal education.

		Rotterdam SFEA	Rotterdam OFEA	Rotterdam SFER	Rotterdam OFER	Rotterdam Total	PBSES Total	PMES Total		
Rotterdam SFEA	r	1								
	*p*									
Rotterdam OFEA	r	0.572	1							
	*p*	0.001								
Rotterdam SFER	r	0.276	0.335	1						
	*p*	0.019	0.004							
Rotterdam OFER	r	0.338	0.693	0.505	1					
	*p*	0.004	0.001	0.001						
Rotterdam Total	r	0.688	0.839	0.707	0.835	1				
	*p*	0.001	0.001	0.001	0.001					
PBSES Total	r	0.247	0.340	0.212	0.429	0.400	1			
	*p*	0.036	0.004	0.074	0.001	0.001				
PMES Total	r	0.362	0.274	0.081	0.285	0.319	0.521	1		
	*p*	0.002	0.020	0.499	0.015	0.006	0.001			
PMES Unrealistic Positive Expectations	r	0.341	0.269	0.067	0.258	0.297	0.459	0.875	1	0.415
	*p*	0.003	0.023	0.575	0.029	0.011	0.001	0.001		0.001
PMES Unrealistic Negative Expectations	r	0.262	0.185	0.070	0.218	0.235	0.415	0.804	0.415	1
	*p*	0.026	0.119	0.560	0.065	0.047	0.001	0.001	0.001	

Pearson correlation analysis, *p* < 0.05, *p* < 0.001.

**Table 8:** The correlation (r) and *p*-values of women who did not receive prenatal education.

		Rotterdam SFEA	Rotterdam OFEA	Rotterdam SFER	Rotterdam OFER	Rotterdam Total	PBSES Total	PMES Total		
Rotterdam SFEA	r	1								
	*p*									
Rotterdam OFEA	r	0.427	1							
	*p*	0.001								
Rotterdam SFER	r	0.520	0.415	1						
	*p*	0.001	0.001							
Rotterdam OFER	r	0.407	0.666	0.516	1					
	*p*	0.001	0.001	0.001						
Rotterdam Total	r	0.722	0.795	0.794	0.832	1				
	*p*	0.001	0.001	0.001	0.001					
PBSES Total	r	0.252	0.451	0.360	0.418	0.475	1			
	*p*	0.031	0.001	0.002	0.001	0.001				
PMES Total	r	0.164	0.232	0.306	0.182	0.285	0.399	1		
	*p*	0.162	0.050	0.008	0.120	0.014	0.001			
PMES Unrealistic Positive Expectations	r	0.084	0.152	0.199	0.112	0.178	0.375	0.874	1	0.281
	*p*	0.477	0.196	0.090	0.341	0.130	0.001	0.001		0.015
PMES Unrealistic Negative Expectations	r	0.203	0.238	0.317	0.198	0.307	0.24	0.712	0.281	1
	*p*	0.083	0.041	0.006	0.092	0.008	0.035	0.001	0.015	

Pearson correlation analysis, *p* < 0.05, *p* < 0.001.

## Data Availability

Dataset available on request from the authors.
